# Successful Treatment for a Large Chronic Expanding Hematoma Treated by Pneumonectomy after Arterial Embolization

**DOI:** 10.1155/2022/1932420

**Published:** 2022-02-18

**Authors:** Aki Fujiwara-Kuroda, Nozomu Iwashiro, Masanori Ohara, Kichizo Kaga

**Affiliations:** ^1^Department of Cardiovascular and Thoracic Surgery, Hokkaido University Graduate School of Medicine, Sapporo, Hokkaido 0608638, Japan; ^2^Department of Thoracic Surgery, National Hospital Organization Hakodate National Hospital, Hakodate, Hokkaido 0418512, Japan

## Abstract

A chronic expanding hematoma is a rare late complication of thoracoplasty for tuberculosis, before the development of drugs. Total resection of a chronic expanding hematoma often requires invasive surgery consisting of combined resection of the lung and chest wall, accompanied by intraoperative bleeding. We report a case of successful surgical resection of a chronic expanding hematoma with preoperative arterial embolization, 48 years after extraperiosteal paraffin plombage for pulmonary tuberculosis. The operative indication or procedure for a chronic expanding hematoma should be determined carefully, and preoperative preparations as well as strategies should be elaborated for safe surgical resection. It is important to share information about treatment for a large chronic expanding hematoma, as we may continue to encounter this complication over the long term.

## 1. Introduction

Chronic expanding hematoma (CEH) is a rare disease that develops slowly, more than a month after surgery, trauma, or epicardial injury [1]. CEH in the thoracic cavity, specifically known as chronic pyothorax, occurs over 20–30 years after pneumothorax, thoracoplasty, or lobectomy for tuberculosis, including extraperiosteal plombage. CEH in the chest has been reported to be one of the late complications of collapse therapy for pulmonary tuberculosis [2] from the 1950s to 1970s in the United States and Japan, and we may continue to encounter this disease in the future. It should be noted that CEH mimics a large malignant tumor. It is difficult to remove CEH due to an adhesion around the capsule of the hematoma as there is a risk of severe complications during surgery. We report a case of successful surgical resection of CEH with preoperative arterial embolization, 48 years after extraperiosteal paraffin plombage for pulmonary tuberculosis.

## 2. Case Presentation

**Figure 1:**
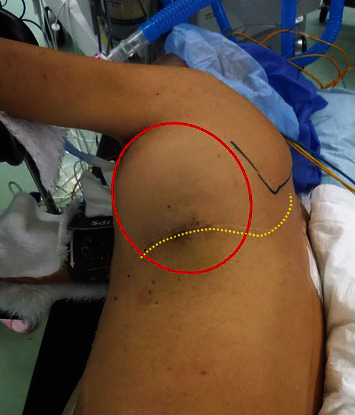
A physical finding. A large mass without redness and tenderness (red circle) is observed in the patient's left chest wall. The yellow dashed line indicates a scar of the posterolateral incision 48 years ago.

**Figure 2:**
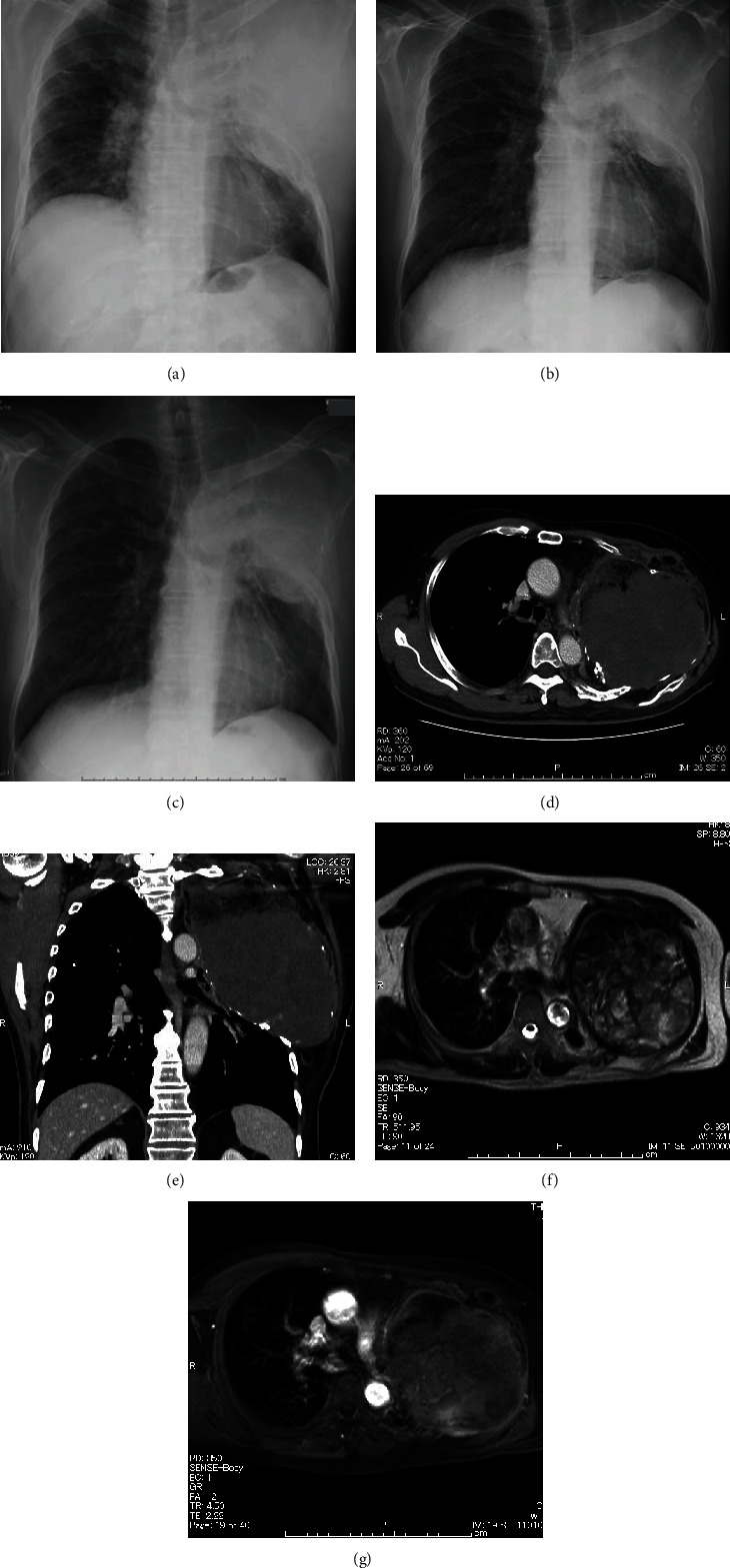
Image findings of (a–c) chest radiography, (d, e) CT scan, and (f, g) MRI. (a) Chest radiography image indicates a large mass from the shoulder to his left upper chest. The size of the tumor did not obviously increase compared to that of (b) 4 years ago and (c) 13 years ago. (d, e) Chest CT scan reveals a 20 cm large mass on the left chest wall with osteoclasis on the ribs and displacing the left lung. It also shows a low-density area in front of the large mass, which indicates paraffin clots from the plombage collapse therapy for tuberculosis 48 years ago. Chest MRI shows a large mass with a high-intensity area in T1-weighted image and homogeneous high- or iso-intensity area in T2-weighted image (f). A limited contrast effect is indicated in the posterior wall of the mass with gadolinium-enhanced MRI (g).

**Figure 3:**
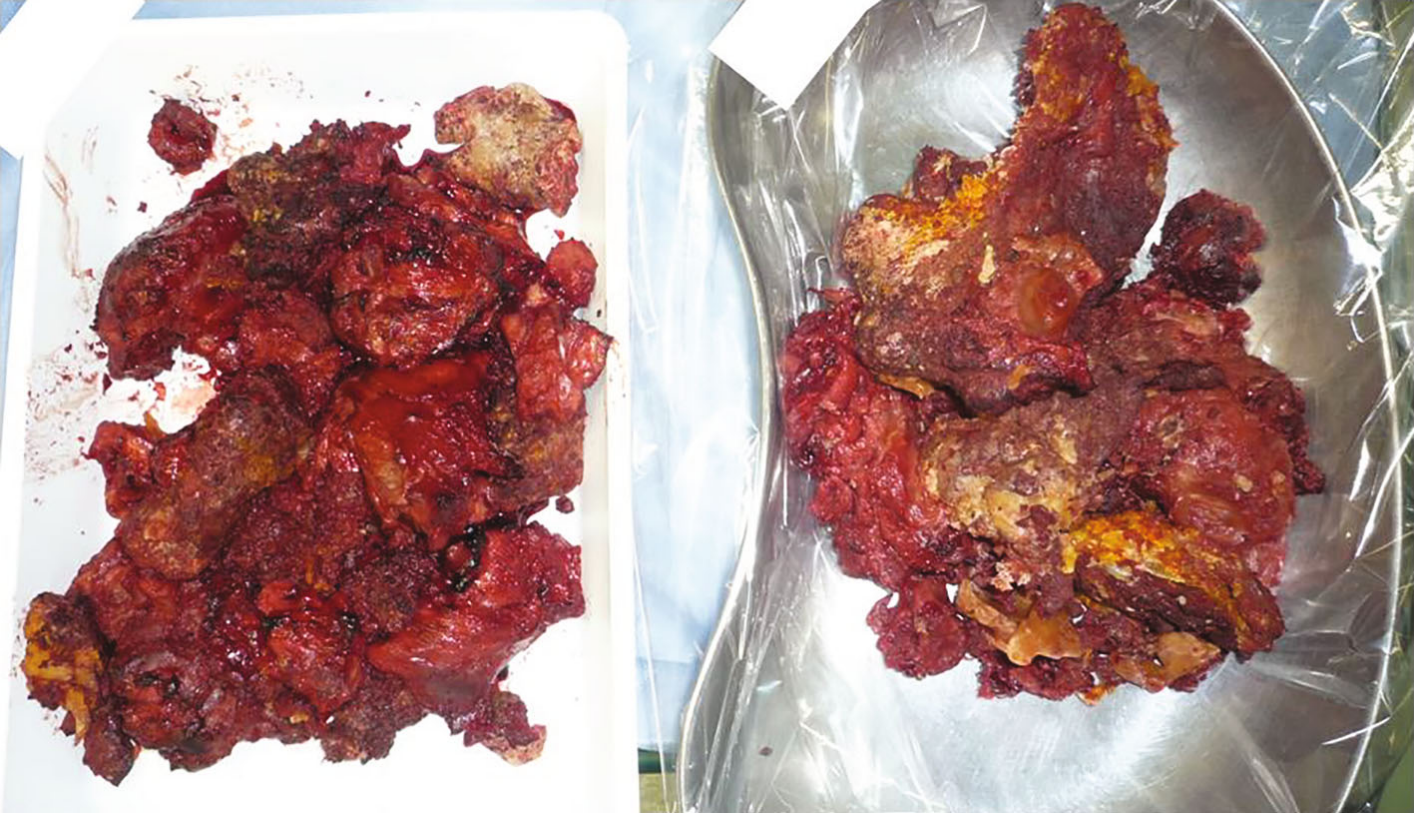
Removed paraffin. There is solid paraffin mixed with necrotic and granular tissues.

A man in his 70s visited our hospital with complaints of pain in his left anterolateral chest wall and numbness without any dermatomal distribution in his left arm for approximately two months. He had a history of cerebral infarction 25 years ago, surgery for tuberculosis in his left lung 48 years ago as standard therapy for tuberculosis at that time, and chemotherapy following relapse 37 years ago. He developed a bulge in his left chest wall, without redness and tenderness (Figure 1), and an orthopedic consultation excluded the possibility of any bone or muscle disorders. Chest radiography (Figure 2(a)) and chest computed tomography (CT) scan (Figures 2(d) and 2(e)) revealed a 20 cm mass in the left chest wall accompanied by calcification. The size of the tumor did not obviously increase compared to that of 4 years ago (Figure 2(b)) and 13 years ago (Figure 2(c)). Following chest magnetic resonance imaging (MRI) (Figures 2(f) and 2(g)), the T1-weighted images revealed that the mass contained a homogeneous high-intensity area and the T2-weighted images revealed a homogenous high- or iso-intensity area. When we performed a percutaneous incisional biopsy under general anesthesia by considering sarcoma, lymphoma, or tuberculous granuloma as the differential diagnosis, some lumps of paraffin were observed beside the mass. In accordance with the tumor biopsy findings related to reactive granuloma, we diagnosed the mass as a CEH caused by extraperiosteal paraffin plombage, which had been performed as a curative surgery for tuberculosis 48 years ago at our hospital. We determined that total resection of the tumor was required to completely relieve the pain. The patient consented to surgery, which was associated with a risk of left pneumonectomy and intraoperative bleeding. We also decided to perform preoperative embolization of the tumor-feeding arteries to minimize intraoperative hemorrhage. He was admitted to our hospital for pain control and preoperative respiratory rehabilitation, and the neuropathic symptoms of pain and numbness were partially relieved by a prescription. The patient was temporarily transferred to another hospital to undergo arterial embolization, and on the 39th day after the biopsy, the left lateral thoracic artery, the left dorsal thoracic artery, three arterial branches of the left internal thoracic artery, and the left 5th intercostal artery, all of which were feeding the tumor, were embolized with gelatin sponge. He returned to our hospital and underwent surgery 6 days after embolization. Posterolateral thoracotomy was performed with the patient in the right lateral decubitus position. We attempted to resect only the hematoma with left upper lobe and to avoid pneumonectomy; however, the left upper lobe was atelectatic due to compression from the large hematoma, and firm inflammatory adhesion made it impossible to identify the interlobar pulmonary artery or preserve the left lower lobe. We unavoidably decided to perform left pneumonectomy along with hematoma removal. We accessed the left main pulmonary artery and the left superior pulmonary vein by incising the pericardium because of adhesion, and combined resection of the second to eighth ribs was also required. The operative time was 386 min, blood loss was 2,270 mL, without uncontrollable bleeding, and he received 560 mL of packed red blood cell transfusion. The resected mass contained necrotic tissues, hemorrhagic materials with capsules, and paraffin lumps (Figure 3). He was transferred from the intensive care unit two days after the surgery, and the chest tube was removed on postoperative day 12. Although he needed long-term rehabilitation to return to daily life as he had no reliable cohabitant, he was discharged from our hospital 3 months after the surgery without any major postoperative complications. Regarding changes in respiratory function, his vital capacity (VC) decreased from 1,750 mL to 1,140 mL, %VC decreased from 56% to 37%, forced expiratory volume (FEV)_1.0%_ decreased from 1,030 mL to 630 mL, and FEV_1.0_/FVC slightly increased from 61% to 68%. No relapse of the tumor or pain was observed 3 years after the resection.

## 3. Discussion

Chronic expanding hematoma (CEH) is a rare disease, as advocated by Reid et al. [1] in 1980. The pathogenesis of CEH is still unclear; however, Labadie and Glover proposed a theory that chronic inflammation due to the breakdown of blood products and increased capillary permeability lead to new bleeding from the capillaries [3]. Although CEH can appear in various parts of the body, it becomes a large mass owing to respiratory movements, beating of the heart, or negative pleural pressure. Repeated microvascularization and hemorrhaging caused by chronic aseptic inflammation expand the space with a hematoma and organized tissues over decades [4].

CEH in the thoracic cavity has been reported as a late complication of collapse therapy for pulmonary tuberculosis, performed before the advent of specific chemotherapy. Extraperiosteal paraffin plombage [5], a collapse therapy, was performed in the 1950s in the United States and in the 1960s and the 1970s in Japan. Paraffin is considered a useful filling because it is less irritating than other materials used for collapse therapy, including gauze, sponge, silk, rubber ball, gelatin, crayons, and acryl balls [4]. Several late complications have been reported in patients who underwent extraperiosteal paraffin plombage 30 to 40 years after the surgery, including CEH, paraffin moving into the subcutaneous tissue, expectoration of paraffin, expanding plombage space, malignant tumors, and infection [2, 4, 6]. In our case, a description was found in written medical records 26 years prior to the surgery, mentioning that he had felt some solid stuff moving in the left chest with a slight pain, although no record before 39 years ago was available. We suppose that this episode was the beginning of the hemorrhage of the plombage space.

The ideal treatment for CEH is complete surgical removal of the hematoma, including the capsule [7]. Failing to totally remove the hematoma or residual capsule can result in a relapse of hemorrhage and hematoma. Total removal of CEH mostly requires massive lung resection, including extrapleural pneumonectomy or pneumonectomy, because of the strong adhesion between the capsule and tissues [8, 9]; therefore, an operative indication should be determined carefully based on the patient's symptoms, performance status, and respiratory function. There have been no reports of changes in respiratory function after CEH surgery. In our patient, the decreases in the VC and %VC were minimized for unilateral pneumonectomy. This is probably because his left lung had been under long-term displacement because he received an extraperiosteal paraffin plombage for pulmonary tuberculosis 48 years ago.

Surgical treatment of CEH is often accompanied by massive bleeding, and preoperative arterial embolization is reportedly beneficial for reducing it [10]. Nakae et al. also reported that preoperative identification of the feeding vessels and severance of blood supply appeared to be useful in preventing CEH recurrence. Moreover, a large mass can become an obstacle for surgeons to securely access the pulmonary vessels that need to be cut. In light of the above, several strategies were planned preoperatively. First, we should attempt to resect only the hematoma cavity; however, it could be difficult due to adhesion with the cavity, chest wall, and left lung. Second, when deciding on combined resection of the lung, left pneumonectomy should be avoided as much as possible, unless inevitable, depending on the adhesion. Third, an intrapericardial approach could be needed to access the left main pulmonary artery or pulmonary veins during lobectomy or pneumonectomy, and changing the position of the patient from lateral to supine could be required for median sternotomy. Finally, we should perform the surgery approximately a week after embolizing the feeding arteries of the CEH, after confirming the absence of complication related to preoperative embolization, including skin or muscle necrosis, neuropathy, cerebral infarction, and allergic reaction. Although changing the body position was unnecessary, other strategies of pneumonectomy, intrapericardial access, and preoperative embolization were necessary.

We report a case of large CEH as a late complication of extraperiosteal paraffin plombage. The operative indications or procedures for the surgical treatment of CEH after thoracoplasty should be determined carefully and closely by considering every possibility based on imaging findings, extent of adhesion, invasiveness, and risks of bleeding caused by adhesion. We believe that a well-considered strategy leads to the successful treatment of CEH.

## Data Availability

All data generated during this study are included in this published article.

## References

[1] Reid J. D., Kommareddi S., Lankerani M., Park M. C. (1980). Chronic expanding hematomas. *A clinicopathologic entity. JAMA*.

[2] Weissberg D., Weissberg D. (2001). Late complications of collapse therapy for pulmonary tuberculosis. *Chest*.

[3] Labadie E., Glover D. (1976). Physiopathogenesis of subdural hematomas. Part 1: histological and biochemical comparisons of subcutaneous hematoma in rats with subdural hematoma in man. *Journal of Neurosurgery*.

[4] Tanaka H., Matsumura A., Iuchi K. (2006). Expanding hematoma after extraperiosteal paraffin plombage. *Surgery Today*.

[5] Fox R. T., Lees W. M., Shields T. W., Iwa T. (1962). Extraperiosteal paraffin plombage thoracoplasty. *The Journal of Thoracic and Cardiovascular Surgery*.

[6] Kuroda A., Ootake S. (2009). A case of paraffin expectoration and pneumonia as a late complication of extraperiosteal paraffin plombage 34 years postoperatively. *Surgery*.

[7] Muramatsu T., Shimamura M., Furuichi M., Ishimoto S., Ohmori K., Shiono M. (2011). Treatment strategies for chronic expanding hematomas of the thorax. *Surgery Today*.

[8] Sakuma T., Takayashiki N., Iguchi K. (2018). Chronic expanding hematoma in the chest: a case report. *Experimental and Therapeutic Medicine*.

[9] Dai W., Zhuang X., Li Q., Xiao P., Shen Y., Zheng P. (2016). Giant chronic expanding hematoma in the chest identified 25 years after a blunt chest trauma. *Molecular and Clinical Oncology*.

[10] Nakae M., Mizoguchi H., Yoshitatsu M., Toda K., Sawa Y. (2019). Successful surgical case of refractory chronic expanding intrapericardial hematoma treated with preoperative coil embolization of the feeding vessels. *Clinical Case Reports*.

